# Exosomes: Emerging Cell-Free Based Therapeutics in Dermatologic Diseases

**DOI:** 10.3389/fcell.2021.736022

**Published:** 2021-10-14

**Authors:** Hui Shi, Min Wang, Yaoxiang Sun, Dakai Yang, Wenrong Xu, Hui Qian

**Affiliations:** ^1^Jiangsu Key Laboratory of Medical Science and Laboratory Medicine, Institute of Stem Cell, School of Medicine, Jiangsu University, Zhenjiang, China; ^2^Department of Clinical Laboratory, The Affiliated Yixing Hospital of Jiangsu University, Yixing, China

**Keywords:** exosomes, cell-free therapy, cell communication, bioengineering, dermatologic diseases

## Abstract

Exosomes are lipid bilayer vesicles released by multiple cell types. These bioactive vesicles are gradually becoming a leading star in intercellular communication involving in various pathological and physiological process. Exosomes convey specific and bioactive transporting cargos, including lipids, nucleic acids and proteins which can be reflective of their parent cells, rendering them attractive in cell-free therapeutics. Numerous findings have confirmed the crucial role of exosomes in restraining scars, burning, senescence and wound recovery. Moreover, the biology research of exosomes in cutting-edge studies are emerging, allowing for the development of particular guidelines and quality control methodology, which favor their possible application in the future. In this review, we discussed therapeutic potential of exosomes in different relevant mode of dermatologic diseases, as well as the various molecular mechanisms. Furthermore, given the advantages of favorable biocompatibility and transporting capacity, the bioengineering modification of exosomes is also involved.

## Introduction

The skin is composed of hair, sweat glands, sebaceous glands and other accessory structures, as well as enriched blood vessels, nerve muscles and lymphatics (Wong et al., 2016). The direct contact with the environment leads to various external and internal damage to it. External factors including sunlight, infection, trauma and chemical media invasion caused by antigen or irritant, and internal factors including genetic factors, psychological factors, medical diseases, drug reactions and so on, which jointly bring significant discomfort to patients in appearance, functionality and even psychology. Some intractable skin diseases and severe burns still lack of effective treatments or control measures. For decades, “cell-based” therapy, especially mesenchymal stem cell (MSC) is holding tremendous promise for tissue regeneration. Emerging evidence shows that the cells like MSCs restore the tissue function mainly by paracrine manner, in which exosomes stand out (Phinney and Pittenger, 2017; Phan et al., 2018). Exosomes shed by MSC are safer and more stable than their donor cells concerning the management of live cells (Nikfarjam et al., 2020). These bioactive vesicles are becoming a leading star in cell-cell communication for delivering message from their cells of origin to the recipients (Wortzel et al., 2019; Li C. J. et al., 2020). Study have indicated that MSC-Exo may emerge as a promising therapeutic approach of SARS-CoV-2 pneumonia and in ischemic diseases (Akbari and Rezaie, 2020; Babaei and Rezaie, 2021). The basis, transformation and application of exosomes derived from stem cells and cells of various skin tissues are springing up and obvious progress has been made (Kalluri and Lebleu, 2020). In this review, the regulatory roles of exosomes in skin differentiation, development and the repair of injury will be further elaborated.

## Brief Description of Exosomes

### Biogenesis and Characterization

**FIGURE 1:**
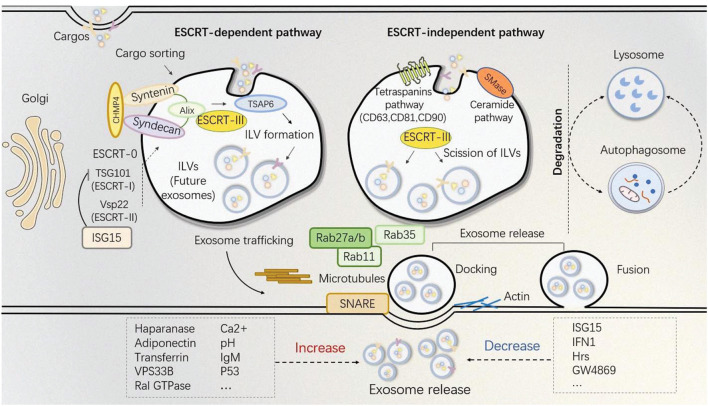
Exosomes biogenesis and release. Cytomembrane lipids and membrane-associated proteins are clustered in micro-domains for MVB formation. Soluble components, such as cytosolic proteins and RNAs are recruited for exosome cargo sorting. Other cargos can be specifically sorted via the trans-Golgi network into MVBs. Mechanisms of exosome biogenesis are well-accepted by different pathways, basically distinguished as ESCRT-dependent and ESCRT-independent manner. Notably, ESCRT-III is required for the final formation of the ILVs, while cargo sorting and membrane budding can be regulated by either pathways. MVBs will either undergo degradation by lysosomes and autophagosome (finally lysosomes) or to dock or fuse with the cytomembrane. Docking and fusion are the two final processes required for exosome release whereby Rab family members, SNARE, as well as actin are involved. Treatments such as haparanase, adiponection, transferrin, VPS33B, Ral GTPase, Ca^2+^, IgM, pH, p53 will help to increase exosome production while ISG15, IFN1, Hrs, GW4869 can block exosome secretion by different mechanisms.

Numerous cells types release exosomes into the extracellular environment after fusing with the cytomembrane. Exosomes are membrane vesicle with a size of 30-150 nm. (Kalluri and Lebleu, 2020). Generally, exosomes can be classified as natural and engineered exosomes. The engineered exosomes are artificially designed and modified while natural exosomes can be natively derived from animals and plants. Ulteriorly, animal-derived exosomes, depending on the normal and tumor culture conditions, consist of two parts, normal exosomes and tumor exosomes (Zhang Y. et al., 2020). The heterogeneity of vesicles which originate from various cells are reflective in density, lipid, protein, density and subcellular origin (van der Pol et al., 2012). Multiple signaling pathways are involved in regulating the exosome biogenesis. Exosomes are acknowledgedly released by microvesicle body (MVB) signaling. At the very beginning, the endosome is formed as a small intracellular body by wrapping little intracellular fluid. The endosome is distinguished as early and late endosomes. After the intraluminal vesicles (ILV) formed, the early endosome develops into the late endosome, namely MVB. The MVB can degrade the contents by fusing with the lysosome or release its ILVs by fusing with the plasma membrane in an exocytotic manner, which are exosomes (Théry et al., 2002; Trajkovic et al., 2008; McAndrews and Kalluri, 2019). During this progress, endosomal-sorting complexes required for transport (ESCRT)-dependent and independent pathways are both involved (Figure 1).

However, after exosomes released, the nomenclature and characterization still not come into a consensus. According to the Minimal Information for studies of Extracellular Vesicles (MISEV), three key areas were addressed: EV isolation/purification, EV characterization and EV functional studies (Lotvall et al., 2014). Regarding the characterization of exosomes, at least three positive protein markers (such as CD9, CD63, CD81, Alix, Hsp70), meanwhile no less than one negative protein marker (such as albumin, calnexin) should be testified. Two different but complementary techniques should be used to characterize single vesicles, such as an electronic or atomic force microscope (which can display near and wide fields) and single particle analyzers (Théry et al., 2018).

### Secretion

The secretion of exosomes is a process in which MVB fusing with the plasma membrane and different mechanisms are involved (Record et al., 2011; Azmi et al., 2013; Beach et al., 2014; He et al., 2018; Yang et al., 2019). Several ESCRT and relevant proteins including Rab, Sytenin-1, TSG101, ALIX, VPS4, actin and SNARE are participants in exosome release (Baietti et al., 2012; Colombo et al., 2013; Friand et al., 2015; Ciardiello et al., 2016; Jackson et al., 2017; Guo et al., 2020). The Rab family proteins regulate the exosome secretion in a large extent, in which Rab27a and Rab27b are widely reported to regulate exosome secretion (Ostrowski et al., 2010). Mutant Rab11S25N inhibiting exosome release confirms that Rab11 is involved in their release (Savina et al., 2002). Rab35 localized on the cell surface of oligodendroglia can regulate the density of exosomes (Hsu et al., 2010). SNARE proteins and synaptotagmin family members also help the docking and fusing of vesicles with the cytomembrane and to release the contained exosomes into the extracellular environment (Beach et al., 2014).

Other alternative ways to affect exosome secretion is certain kind of inducement or stimulation of the origin cell. An increase of intracellular Ca^2+^ and activation of protein kinase C promotes both the generation and delivery of exosomes (Nguyen et al., 2016). In cancer cells, the acute elevation of calcium also causes a quintuple increase in the amount of exosome^ALIX+CD63+CD9+^ (Messenger et al., 2018). Moreover, Transferrin (Tf)-stimulated exosome release is in a calcium ion -dependent manner (Savina et al., 2003). Heparanase involves in almost every related step from biogenesis to release of the exosomes as well as regulating the exosomal protein cargo (David and Zimmermann, 2020; Purushothaman and Sanderson, 2020). Diabetic sera are also found to affect exosome release by disrupting the normal MSC-derived exosome signaling pathway *in vitro* (Rezaie et al., 2018b) (see more stimulation methods in Table 1). Though some different mechanisms or ways of exosome biogenesis and release are discussed currently, they are not all-inclusive and there still remains plenty room for fully uncovering exosome pathways in all situations.

**TABLE 1:** Approaches to affect exosome release.

Agent	Mechanisms	Models	Production	References
Rab27a/Rab27b	Multivesicular endosomes docking		increase	Ostrowski et al., 2010
Rab11	Rab11S25N mediated exosome release	K562	increase	Savina et al., 2002
Rab35	GTP-dependent localization	Oligodendroglia cells	increase	Hsu et al., 2010
Ca^2+^	a. Activation of protein kinase C b. .Munc13-4/Rab11 dependent pathway	Human RBCs MDA-MB-231	increase	Nguyen et al., 2016; Messenger et al., 2018
PH	a. Low PH induced high rigidity and sphingomyelin content b. .MVB acidification	Melanoma cells Human cells	increase	Parolini et al., 2009; Sung et al., 2020
Transferrin	Ca^2+^ dependent manner	K562	increase	Savina et al., 2003
P53	a. Regulation of TSAP6 b. .IGF-1/AKT/mTOR signaling	BMDCs cells respond to stress	increase	Lespagnol et al., 2008; Feng, 2010
IgM	BCR activation	CLL cells	increase	Yeh et al., 2015
Haparanase	Accumulation of syndecan-1	MCF-7	increase	David and Zimmermann, 2020
Adiponectin	Binding to T-cadherin	MSCs	increase	Nakamura et al., 2020
VPS33B	GD12/Rab11a/Rab27a pathway	HSCs, LICs	increase	Gu et al., 2016
RaI GTPase	colocalization with SYX-5	nematodes, mammary tumor cells	increase	Hyenne et al., 2015
ISG15	Targeting TSG101 mediated plasma membrane formation	HEK293	decrease	Nabhan et al., 2012; Choudhuri et al., 2014
IFN1	ISG15 triggered MVB-lysosome fusion	HEK293T	decrease	Villarroya-Beltri et al., 2016
GW4869	nSMAse-independent mechanism	myeloma cells SKBR3 cells	decrease	Stoffel et al., 2016; Menck et al., 2017; Vuckovic et al., 2017
				

### Transporting Cargos

Transporting cargos in their lipid bilayer membrane, exosomes successfully pass on the message from parent cells to the recipient cells, which includes proteins, nucleic acids and lipids (Tkach and Théry, 2016; van Niel et al., 2018). The mechanisms of how exosome uptake by target cells remains unclear (Villarroya-Beltri et al., 2014), it is more likely to be a dynamic process. Exosomes may enter cells directly through different mechanisms like the participation of calveolae, clathrin, lipid rafts, direct fusion with cell membrane, receptor-ligand interaction, endocytosis by phagocytosis and also micropinocytosis (Kalluri and Lebleu, 2020). However, at the meantime, the endocytosis process also gives rise to the *de novo* formation of exosomes by cells. Whether the release of exosomes which generate endogenously or take up occurs separately or together is still hard to tell.

Previously, exosomes are considered as vehicles transporting cargos to discard and unnecessary for their cells of origin, but now accumulating evidence confirms that these vesicles as well as their contents are playing full roles in the physiological and pathological process (Phinney and Pittenger, 2017; Bebelman et al., 2018; van Niel et al., 2018; Kalluri and Lebleu, 2020). The comprehensive exosome-mediated crosstalk between different cell types are now attracting increasing attention, exosomes are recognized as a new “cell-free” therapeutic strategy in regenerative medicine and the roles of exosomes in skin development and cutaneous wound healing will be discussed in detail below.

## Exosomes and Dermatologic Disease

**FIGURE 2:**
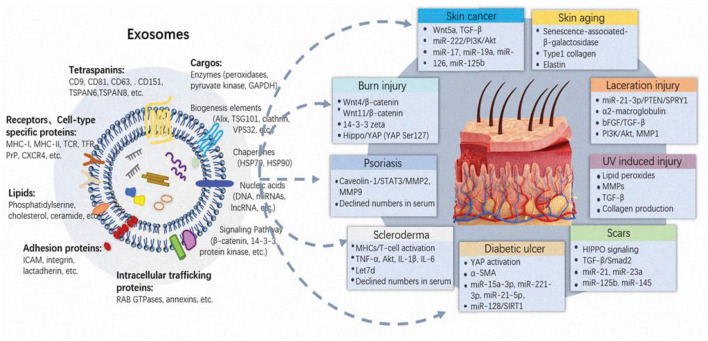
Main composition of exosomes and their functions on skin injuries. Despite the different origin and mode of biogenesis, exosomes display a similar appearance, and common composition. Exosomes are featured by tetraspanins, receptors or cell-type specific proteins, lipids, adherin proteins, intracellular trafficking proteins. Deep analysis of extracellular vesicle composition reveal that they convey various cargoes, including nucleic acids, proteins (biogenesis elements, chaperones and signaling pathway molecules) and lipids, which all vary widely between cells and conditions. The vast information will directly affect the fate and function of exosomes in skin injuries. Exosomes participate in multiple cutaneous diseases including laceration, burn, aging, diabetic wound healing, scar formation, skin autoimmune disease and skin cancers, through different biological pathways.

Under pathological conditions like diabetes or severe burns, skin function is not fully restored via the normal wound healing process, leading to potentially serious complications such as ulcers or infections (Nuschke, 2014). Therefore, it is crucial to complete wound healing with the least delay after injury. Stem cells, particularly MSCs, present great clinical application prospects in skin tissue regeneration for their advantages of extensive sources, easy expansion *in vitro* and low rejection after transplantation *in vivo*. It is well accepted now cells like MSCs releasing repair promoting factors in a paracrine manner. Paracrine is the dominating way to facilitate damage repair, whereby exosome outstands (Phinney and Pittenger, 2017; Mehryab et al., 2020; Rahmani et al., 2020). The findings open up the alternative avenue of cells such as MSCs to exosomes for safer, potent and “cell-free” based therapeutic application. A good deal of preclinical experiments has confirmed the multiple roles of exosomes in various skin injury repair (Figure 2 and Table 2).

**TABLE 2:** Functions of exosomes derived from different origin in cutaneous disease.

Origin	Recipient	Mechanisms	Functions	References
hucMSC	fibroblasts and endothelial cells	miR-21-3p/PTEN/SPRY1	promote proliferation and migration	Hu et al., 2018
hucMSC	fibroblasts	enriched of alpha-2-macroglobulin	improve the activity and migration ability of skin fibroblasts	Bakhtyar et al., 2018
BM-MSC	dermal vascular endothelium	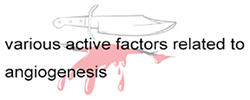	formation of new blood vessels	El-Tookhy et al., 2017
AdMSC	fibroblasts	bFGF and TGF-β PI3K/Akt signaling pathway	increased proliferation and migration	Zhang W. et al., 2018
autologous human epidermal cells	dermal fibroblasts	activating matrix metalloproteinase-1	cell proliferation and cause the degradation extracellular matrix	Zhao et al., 2017
hucMSC	dermal fibroblasts epidermis cells	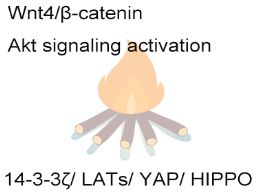	promote the proliferation of skin cells re-epithelialization of epidermis orchestrate the distribution collagen Enhance angiogenesis	Zhang et al., 2015a, 2016
hucMSC	dermal fibroblasts epidermis cells		restrict the scar formation	Zhang et al., 2016
hucMSC	fibroblasts	miR-21, miR-23a, miR-125b and miR-145 targeted TGF-β/SMAD2	reduce the expression of α-SMA and collagen deposition control scar formation	Fang et al., 2016
hiPSC	human dermal fibroblasts	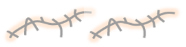	protective and anti-aging effect	Oh et al., 2018
human umbilical cord blood	epidermis cells	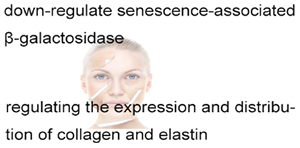	a certain degree of anti-aging effect	Kim et al., 2017
iPSC	human dermal fibroblasts	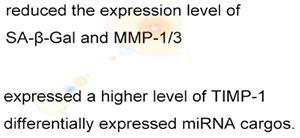	protect skin from ultraviolet damage	Oh et al., 2018
3D fibroblasts spheroids	not mentioned		inducing collagen synthesis	Hu et al., 2019
Platelet-rich plasma	endothelial cells and fibroblasts	YAP activation	regeneration of chronic skin wounds in diabetic rats	Guo et al., 2017
iPSC	fibroblasts	not clear yet 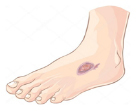	accelerates diabetic wound healing	Kobayashi et al., 2018
gingival MSC	not mentioned	not clear yet	promote the healing and nerve repair	Shi Q. et al., 2017
melanoma cell	MDSC lymphatic endothelial cells	enhance the tolerance capacity of lymph gland to tumors	immune evasion	Hood, 2016
melanoma cell	skin cells	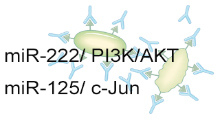	tumor migration and progression	Alegre et al., 2014; Felicetti et al., 2016
SCC cell	microenvironment cells	TGF-β activation	tumor growth and metastasis	McAndrews and Kalluri, 2019
donor MSC	recipient BM-MSC	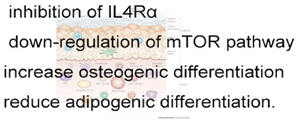	caused the rescue of osteopenia impaired BM-MSCs, tight skin, and immune disorders in scleroderma mice	Chen et al., 2017

### Exosomes and Laceration

Exosomes present favorable repair activity in skin lacerations and full-layer resection. After partial resection of back skin in 12-week-old C57B/6 mice, human umbilical cord MSC derived exosomes (hucMSC-Ex) effectively promote wound angiogenesis and epidermal regeneration *in vivo*. Further exploration shows that the mechanism is related to miR-21-3p conveyed by exosomes, which regulates cell function by inhibiting its downstream PTEN and SPRY1 target genes (Hu et al., 2018). Protein spectrum reveals that hucMSC-Ex is enriched in a large amount of alpha-2-macroglobulin improve the activity and migration ability of skin fibroblasts (Bakhtyar et al., 2018). Bone marrow MSC (BM-MSC) derived exosomes can accelerate the healing of wound by promoting the neovascularization in the dermal laceration model of large animal dogs, and the mechanism of this repair effect is also related to the fact that BM-MSC exosomes contain various active factors related to angiogenesis (El-Tookhy et al., 2017). Adipose-derived MSC (AdMSC) exosomes are internalized and ingested by skin fibroblasts to accelerate the proliferation and migration of target cells. Expression levels of repair related factors such as collagen, bFGF and TGF- beta are increased, and the PI3K/Akt signaling pathway is activated by AdMSC exosomes in damaged skin cells to accelerate the repair process (Zhang W. et al., 2018). Additionally, dermal fibroblasts can also internalize autologous human epidermal cells derived exosomes in order to promote the proliferation of cell and cause the degradation of some extracellular matrix by activating matrix metalloproteinase-1(MMP-1). Exosomes derived from human epidermal cells are applied in the full-layer skin excision model of rats, whereby the epidermal regeneration is basically completed within two weeks, which also have significant healing effect (Zhao et al., 2017).

### Exosomes and Burn

**FIGURE 3:**
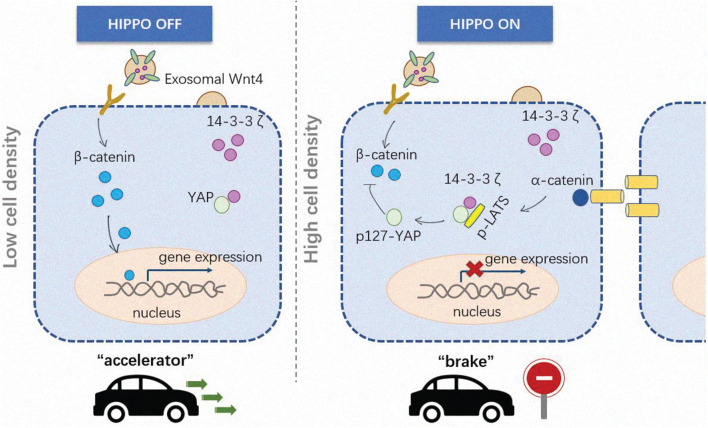
The dynamically regulatory role of exosomes in burn injury. In the early stage of repairing, cells are in a low density, exosomal Wnt4 activates β-catenin in skin cells to promote proliferation and migration, meanwhile stimulates vascular endothelial cell to enhance angiogenesis. With the progression of wound healing, cell density becomes from low to high, the cell-cell contact triggers the activation of Hippo signaling, leading to p-LATS accumulation. 14-3-4ζ released by exosomes works like a “table” to facilitate the combination of YAP and LATS, so as to accelerate the phosphorylation of YAP, the phosphorylated YAP shows a intracellular retention rather than nuclear translocation to suppress the down-stream gene expression and finally inhibit the cell proliferation and migration. The exosomes mediated dynamic regulation in different stage of wound healing, works as an “accelerate” to promote wound healing in early stage, while as a “brake” to prevent excessive proliferation and scar formation.

Burns is a common injury in daily life, which occurs in 50 to 100 cases per 10,000 people annually (Lewis, 2013). At present, the treatment of large-scaled scald is mainly through systemic support, wound cleaning, scab excision and surgical skin grafting (Calota et al., 2012), which are difficult to grasp the scab depth and easy to be infected, and in source tension of skin grafts (Eisenbeiß et al., 2012; Lootens et al., 2013; Nacer Khodja et al., 2013). The skin deep burn and scald within 3 weeks is difficult to repair completely by itself, and is easy to form scar tissue (Cuttle et al., 2006; Ribeiro et al., 2009). Thus, shortening the wound healing time is particularly important. In our previous study, we successfully established the deep secondary burn injury model in SD rats, and found that hucMSC-Ex accelerated the cutaneous wound recovery by facilitating the proliferation of skin tissue cells and the re-epithelialization of epidermis, as well as orchestrating the rational distribution of collagen in skin tissue and enhancing angiogenesis (Zhang et al., 2015a,b). Interestingly, long-term (more than 4 weeks) observation showed that hucMSC-Ex conversely inhibited the proliferation of skin cells and the activation of β-catenin, meanwhile, hucMSC-Ex restricted the amplification of skin stem cells, the expression of α-SMA in dermal cells and the deposition of collagen III. Along with the healing process, the cell density varies from low to high, which triggers the Hippo signaling activation. Mechanically, hucMSC-Ex could deliver 14-3-3 zeta protein to combine YAP and LATs, thus speeding up the phosphorylation of YAP to limit the continuous activation β-catenin (Zhang et al., 2016). The dynamic regulation pattern prevents excessive hyperplasia of skin tissue and scar formation after injury (Figure 3).

### Exosomes and Scars

During the proliferation period of skin injury repair, fibroblasts, epidermal cells and vascular endothelial cells jointly promote the formation of vascular network, and in the meantime, cells migrate from the edge to the surface of the wound, reducing the area of the wound surface to promote the smooth progress of epidermal regeneration. In this process, excessive activation and recruitment of fibroblasts under pathological conditions often lead to scar formation and fibrosis (Wynn and Ramalingam, 2012; Van De Water et al., 2013). In our studies, hucMSC-Ex can inhibit tissue over-proliferation and scar formation by activating Hippo signaling pathway in the later stage of burn injury repair, providing a guarantee for the security of exosome *in vivo* (Zhang et al., 2016). In addition, it is worth noting that the TGF-β family plays a positive regulatory role in cell differentiation, proliferation and metabolism during tissue injury and repair, while the pathological continuous activation of TGF-β activates the SMAD2 signaling pathway, which can easily lead to fiber deposition and scar formation (Frei et al., 2015). HucMSC-Ex could effectively inhibit the activation of fibroblasts in mice after full-layer resection of dorsal skin. A key group of miRNAs, miR-21, miR-23a, miR-125b and miR-145, could inhibit the activation of fibroblasts by targeting the TGF-β/SMAD2 signaling pathway, reduce the expression of α-SMA and collagen deposition, so as to control scar formation (Fang et al., 2016). At the same time, researchers also propose to conduct gene transfection modification on hucMSC-Ex based on this group of miRNA molecules, so as to acquiring a better and safer therapeutic strategy.

### Exosomes and Aging

Skin aging is mainly for the genetic factors and easily found in exposure and the exposed parts, with wrinkles and sagging as the main characteristics. Pathologically, the skin epidermis and the dermis thickness become thinner, dermal collagenous fibers and collagen content declines with neatly arranged collagen bundles, clinically characterized by skin with fine wrinkles. hiPSC exosomes are used to confront aging skin, the expression of senescence-associated-β-galactosidase, a natural aging marker, is significantly down-regulated, and the expression of type I collagen in aging fibroblasts increased, thus playing a good protective and anti-aging effect on human dermal fibroblasts (Oh et al., 2018). Human umbilical cord blood derived exosomes penetrate into the epidermal layer of skin within 15 h, and reach a certain degree of anti-aging effect by regulating the expression and distribution of collagen and elastin in skin cells (Kim et al., 2017) and MSCs derived exosomes can also ameliorate aging and age-related diseases (Ahmadi and Rezaie, 2021).

### Exosomes and Ultraviolet Damage

Ultraviolet damage of skin is the accumulation of ultraviolet damage in the process of skin aging, which is the result of the interaction of endogenous aging and ultraviolet radiation, featured tissue disintegration, fragmentation and collagen bundle dispersion (Bellei et al., 2018). Matrix metalloproteinases (MMPs) specifically degrade almost all extracellular matrix (ECM) components and play a vital role in photoaging of skin. Photoaging is found to gradually impair the function of human dermal fibroblasts (HDFs), which generate collagen and matrix metalloproteinase in dermis (Jeong et al., 2015; Zorin et al., 2017; Hu et al., 2019). The iPSCs-Ex can increase the expression level of collagen type I in the photo-aged HDFs and reduce the expression level of SA-β-Gal, MMP-1/3 and restore the collagen type I expression in senescent HDFs (Oh et al., 2018). Another interesting research compared exosomes derived from monolayer culture of HDFs (2D HDF-XOs) with that from the three-dimensional spheroids (3D HDF-XOs). The result showed that there was a notably higher level of TIMP-1 and differentially express miRNAs in 3D HDF-XOs (Hu et al., 2019). 3D HDF-XOs mainly work through the downregulation of TNF-α and the upregulation of TGF-β to induce collagenation and antiaging. Additionally, an interdisciplinary study showed that marine sponge spicules (SHSs) significantly enhanced the skin targeted delivery of hucMSC-Ex, which also revealed anti-photoaging effects in mice, including reducing micro-wrinkles and enhancing the expression level of extracellular matrix, and facilitating the skin to recover shortly (Zhang K. et al., 2020).

### Exosomes and Diabetic Wound Healing

Diabetic foot ulcer (DFU) is one of the most common and major complications of diabetes. At present, the pathogenesis of DFU is considered to be the result of the interaction between peripheral blood vessels and peripheral neuropathy. The complexity of the pathogenesis and the difficulty of wound healing make it one of the problems that threaten the life and health of diabetic patients (Iversen et al., 2009; Goodall et al., 2020). Our previous research has confirmed that hucMSC-Ex can alleviate insulin resistance in type 2 diabetic individuals, increase the distribution of GLUT on the surface of liver, muscle, fat and other tissue membranes, and improve the amount and activity of glucose metabolism enzymes in tissues such as liver, muscle and fat, promote the body’s intake and utilization of glucose in the blood to reduce blood sugar in individuals with type 2 diabetes. Specific mechanisms such as exosome directly carrying GLUT molecules to receptor cell (liver, muscle, and fat) membranes, which facilitate extracellular glucose transportation, provide a basic mechanism for exosomes to treat diabetes-related complications (Sun et al., 2018). Conversely, study indicated that diabetes could decrease angiogenic and restorative effect of stem cells *in vitro* (Rezaie et al., 2018a). In diabetic mice, human fibroblast-derived exosomes can promote the acceleration of wound repair (Geiger et al., 2015). The platelet-rich plasma-derived exosomes can activate YAP and promote the regeneration of chronic skin wounds in diabetic rats (Guo et al., 2017). iPSC exosomes enhance fibroblast migration and proliferation, accelerates wound healing and local nerve regeneration in diabetic foot (Kobayashi et al., 2018). The gingival MSC exosome and the synovial MSC exosome have also been reported to promote the healing and nerve repair of diabetic ulcer wounds, however, the specific mechanism requires to be refined (Shi Q. et al., 2017).

### Exosomes and Skin Cancer

Exosomes are closely related to tumorigenesis, such as U87 MG human glioblastoma, colorectal cancer, skin cancer (Ahmadi et al., 2021; Soraya et al., 2021). Study showed that exosomal cargos modulate autophagy and play pivotal roles in neoplasis, tumor heomostasis and metastasis (Hassanpour et al., 2020; Jafari et al., 2020; Salimi et al., 2020). Exosomes in skin cancer microenvironment are secreted and enriched, provide cancerous cell the platform to metastasize and attacking the host’s immune system (Gowda et al., 2020). Melanoma is the most malignant of skin neoplasms, and melanoma cell-derived exosomes affect the sentinel lymph node genes in a variety of ways to promote tumor progression. In melanoma cells, Wnt5a promotes the release of immunoregulatory and proangiogenic factors from exosomes (Ekström et al., 2014), while melanoma cell-derived exosomes can regulate lymphatic endothelial cells and myeloid-derived suppressor cells (MDSCs) to enhance the tolerance capacity of lymph gland to tumors, finally achieving immune evasion (Hood, 2016). Metastatic melanomas release exosomes carrying PD-L1, which also be able to suppress CD8^+^T cells function and facilitates tumor growth (Chen et al., 2018). Besides, exosomes can also contain miRNAs, such as miR-222, activates PI3K/AKT signaling pathways in skin cells to promote tumorigenesis (Felicetti et al., 2016). Human metastatic melanoma serum exosomes show a high expression level of miRNA-17, miRNA-19a, miRNA-126 (Pfeffer et al., 2015). MiRNA-125b is selectively expressed by tumor cells due to its ability to inhibit tumor-associated c-Jun protein expression (Alegre et al., 2014). Due to tissue-specific miRNAs can be encapsulated in EVs and distributed in multiple biological fluids, they may serve as a tumor biomarker in neoplasia early progression (Jafari et al., 2019).

In squamous cell carcinoma (SCC), exosomes also serve as an important vehicle for tumor and microenvironment communication, and reactivate tumor-associated signals such as TGF-β in order to promote metabolism and growth of cancer cells (McAndrews and Kalluri, 2019).

### Exosomes and Other Skin Lesions

Scleroderma is characterized by inflammatory, degeneration, thickening, and fibrosis, which in turn is hardened and atrophied, thus causing multiple system damage. Studies have reported a significant reduction in exosome levels in peripheral blood serum from patients with scleroderma, which is highly likely to be associated with impaired exosome transport system caused by vascular abnormalities (Nakamura et al., 2016a). This decrement of exosome production in scleroderma patients’ serum may be attributed to the disordered transport of exosomes which is from the skin epithelium to the blood flow, resulting in the down-regulation of collagen I that delays wound repair, leading to a higher susceptibility to cutaneous ulcer (Wermuth et al., 2017). Recently, it has been reported that miR-151-5p is a diagnostic and therapeutic potential biomarker of scleroderma. Chen et al. (2017) found that exosomal miR-151-5p released from donor MSCs could inhibit the expression level of IL4Rα and suppress the activation of mTOR pathway, which leads to an increase in osteogenic differentiation and decrease in adipogenic differentiation. Further, miR-151-3p delivered systemically protected the scleroderma mice from immunologic derangement, tight skin, osteopenia and impaired bone marrow MSCs (Chen et al., 2017). Wermuth et al. (2017) found that exosomes derived from scleroderma patients cell culture supernatant were associated with the stimulation of genes expression, including collagen type III alpha 1 chain, COL1A1 and fibronectin-1 encoding ECM components (Chen et al., 2017). In profiles of neutrophils exosomes derived from scleroderma patients, 281 dysregulated lncRNAs and 22 dysregulated miRNAs are identified as well as further analysis were conducted of their target genes and downstream signaling pathways, which could be potential therapeutic targets and biomarkers in scleroderma (Li L. et al., 2020).

Psoriasis is an autoimmune disease of the skin, often accompanied by excessive proliferation of keratinocytes, while iron reserves often affect the working state of the patient’s immune system. Some researchers have found that important proteins regulating iron reserve such as hepcidin and soluble transferrin receptor are at low expression levels in serum exosome of patients with psoriasis, presumably affects immune function and thus affects the course of the disease (El-Rifaie et al., 2018).

## Exosomes Participate in Various Skin Damage Repair Mechanisms

### Coagulation

Exosomes can be extracted from saliva, mononuclear macrophages, and platelet secretions. After skin trauma, obstruction of coagulation function affects the repair time of wounds, and the blocked coagulation function is relevant to the change of tissue factor (TF) conformation or the presence of TF inhibitory molecules. Saliva-derived exosomes can shorten wound clotting time by transporting active TF in combination with factor VII. At the same time, these different sources of exosomes secreted into the intracellular space can also activate coagulation by transmitting TF, for example, exosome secreted by monocytes can bind to platelets and release their own TF into platelets to activate downstream cascades to promote thrombin and fibrin clot formation (Biró et al., 2003; Del Conde et al., 2005).

### Proliferation

Proliferation of skin cells plays a vital role in skin damage repair. At present, exosomes from various tissue and cell regulate the proliferation of skin cells, such as MSC exosomes (Du et al., 2014; Shabbir et al., 2015; Jiang et al., 2020), mouse ESC exosomes (Jeong et al., 2014), endothelial progenitor cell-derived exosomes (Li et al., 2016) and fibroblast-derived exosomes. These exosomes functioning in regulating cell proliferation in two ways, activate cell cycle related genes or increase growth factor expression levels. The exosomal RNAs or proteins are delivered to the target cell, such as Wnt4 transportation to motivate the β-catenin signaling pathway in the epidermal cell (Zhang et al., 2015a), or delivering the small RNAs to target the downstream Akt, Erk, MAPK signaling pathway, etc. (Zhang et al., 2016; Guo et al., 2017), ultimately attributed to the regulation of cell cycle-related genes such as cyclinA1, cyclinD1, cyclinD3 to promote cell proliferation. Additionally, exosomes can also activate interleukin-6 (IL-6), insulin-like growth factor-1 (IGF-1), vascular endothelial growth factor (VEGF) and other growth factors, which stimulate damaged skin cells proliferation. The proliferative effect of exosomes can be caused both by paracrine affecting surrounding cells and autocrine to *de novo* affect itself.

### Migration

Proliferation and migration of cells are pivotal for wound healing. With the onset of the clotting period, immunoinflammatory cells such as macrophages and neutrophils are first recruited to the damaged site to get rid of necrotic tissue fragments as well as phagocytose infected microbe. Subsequently, epidermal cells and dermal fibroblasts begin to migrate to the injured sites to fill the vacancies and promote wound healing (Singer and Clark, 1999). In this process, exosomes from various cells are involved in the regulation of cell migration, such as MSCs, keratinocytes, endothelial cells and fibroblast-derived exosomes (Cheng et al., 2008). Keratinocyte-derived exosomes can promote their own migration by transporting heat shock protein 90α (HSP90α) in an autocrine manner. In addition, keratinocyte-derived exosomes can also promote the migration of vascular endothelial cells, thereby promoting the regeneration and repair of blood vessels (Leoni et al., 2015). Moreover, keratinocyte-derived exosomes enhance the migration of dermal fibroblasts by transporting HSP 90α, which mechanically relate to its interaction with cell surface receptor LRP-1/CD91 (McCready et al., 2010). MSC exosomes regulate fibroblast migration by activation of Erk1/2 signaling pathway, resulting in up-regulating the expression of IL-6, cyclin1, MMP-1, N-cadherin and other factors in target cells (Huang et al., 2015; Hu et al., 2016).

### Vascularization

Angiogenesis provides oxygen for repairing tissues and supply nutrients. The proliferation of vascular endothelial cells is an essential constituent of it. Moreover, it also requires interaction with angiogenic, endothelial cells and the surrounding extracellular matrix environment (Liekens et al., 2001). The wound area exhibits chemotactic effect on vascular endothelial cells, enabling endothelial cells to penetrate the vascular basement membrane, enter the extracellular matrix, form a lumen-like structure and continuously extend and branch, and finally generate a new vascular network. In this process, human MSC and endothelial cell-derived exosome can increase the density of neovascularization in the wound area, which is related to the promotion of endothelial cell proliferation and migration. In addition, endothelial progenitor cells and platelet-rich plasma-derived exosomes can activate the expression of pro-angiogenic genes in target cells, such as IL-8, IL-6, fiber growth factor 2, E-selectin, etc. The expression of these factors will further activate Erk1/2 and other related signaling pathways, activate downstream VEGFA, VEGFR-2, c-Myc, cyclinD1 and other target genes, thereby further promoting neovascularization (Hromada et al., 2017). In our previous studies, we found that hucMSC-Ex repaired wounds by promoting Wnt4/β-catenin signaling pathway in vascular endothelial cells (Zhang et al., 2015a). Conversely, some researchers find exosomes to inhibit the formation of blood vessels by binding to CD36 on the surface of target cells (Ramakrishnan et al., 2016). Therefore, despite of a great potential in regulating angiogenesis, it is still necessary to specifically analyze the source, type and specific pathological conditions when exosomes are used.

### Skin Stem Cell Mobilization and Participation

Stem cells are the important reason to explain the super self-repair ability of skin. Epidermal stem/progenitor cells generally locate in the basal layer of the epidermis to meet the daily keratin renewal metabolism or the need to fulfill new cells after skin damage. The proliferation and differentiation of skin stem cells are affected by multiple factors, and finally achieve the balance between proliferation and differentiation (Li and Sen, 2016; Yang et al., 2020). In the model of deep second-degree scald repair in rats, we confirm that hucMSC-Ex can activate CK19 and α6 integrin-positive epidermal stem cells in the early stage of repair, and make the epidermal stem cells return to resting state after the repair is completed. The mechanism may be related to the dynamic regulation between Wnt/β-catenin signaling pathway and Hippo/YAP signaling pathway (Zhang et al., 2016). In addition, epidermal stem cells are involved in epidermal regeneration in the early post-traumatic period, but do not play a role in late and normal epidermal renewal. Exosomes from autologous stem cells are also effective in promoting proliferation, angiogenesis and wound healing of damaged skin cells (Lu et al., 2019).

### Immunoregulation

After skin trauma and infection, a great quantity of immune cells are recruited to the lesion region, Exosomes transport pathological nucleic acids, proteins and lipids, and take part in antigen-promoting, immune response-related receptors activation, and defense induction, which therefore arouse interests to consider exosomes as good immune-based therapeutics and vaccine candidates (Schorey and Harding, 2016).

**FIGURE 4:**
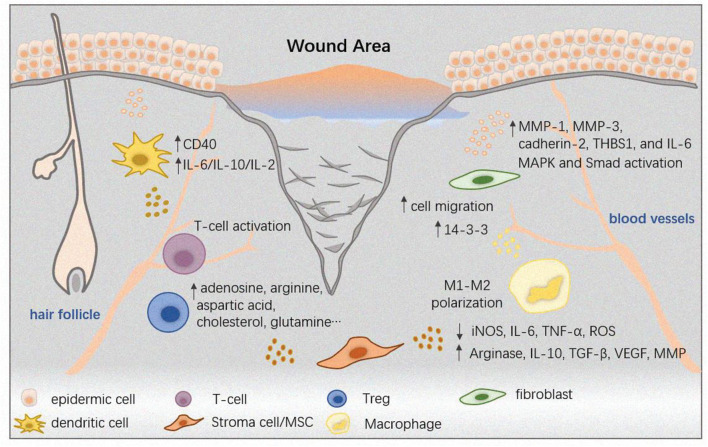
The immunoregulatory roles of exosomes in skin diseases. The skin relies on the immune system for essential functions, such as thermoregulation and infection control. The wound healing process is a complex one with at least three stages, while immune cells participate in them all. Neutrophils and macrophages infiltrate into the wound healing as soon as the inflammatory stage begins. The wound area is a complicated microenvironment that cells inside of it like epidermic cells, fibroblasts, stroma cells can interact with immune cells including T-cells, Tregs, dendritic cells and macrophages by releasing specific bio-molecules, jointly affect the fate of disease.

Skin and immune cells mediate the corresponding immunological effects by secreting exosomes. Keratinocytes expedite the stimulation and maturation of dendritic cells by secreting exosomes, enhance the expression of CD40, and produce a large number of cytokines such as IL-6, IL-10, IL-12 (Kotzerke et al., 2013). Exosomes from circulating immunological cells such as monocytes can also promote the expression of MMP-1 in dermal fibroblasts by transporting 14-3-3 protein, thereby promoting cell migration to the wound area (Medina and Ghahary, 2010). Moreover, MSC exosomes are involved in autologous skin transplantation by regulating the inhibitory immune cell Treg (Zhang B. et al., 2018; Showalter et al., 2019), and exosomes from sweat can also regulate skin immunity, which is the donor exosomes trigger alloreactive T cell responses after transplantation (Marino et al., 2016). Furthermore, the M2 Macrophage-derived exosomes (M2-Exo) induce M1-M2 conversion *in vitro* to promote wound healing by facilitating neovascularization, collagen deposition as well as re-epithelialization (Kim et al., 2019). In recent years, immune cell such as dendritic cell-derived exosomes are used to stimulate T cells to activate host anti-tumor immunity, and have entered Phase I and Phase II clinical trials (Besse et al., 2016; Figure 4). Conversely, some studies have found that after skin transplantation, some donor-derived exosomes unexpectedly trigger an immune response and attack receptor tissue cells by activating autologous T-cell immunity, which reminds us the immune system in skin is quite a complex network that every type of cells inside is possibly to communicate with other cell types nearby or in a distance, some of the “cross-talk” mechanisms are unlocked while some are not, the function and application of exosomes in immune regulation remains to be more fully lucubrated (Morelli et al., 2017; Benichou et al., 2020).

### Extracellular Matrix Remodeling

Exosomes are involved in the hemorrhagic, inflammatory and proliferative phase of skin damage repairing as well as the tissue remodeling. Exosomes from various cells have shown an effective regulatory function on extracellular matrix. Elastin is an important component of the extracellular matrix maintenance structure, and exosomes can promote elastin expression during tissue remodeling (Zhang et al., 2015c). Collagen mainly secreted by fibroblasts also takes part in tissue remodeling. Adipose-derived MSC exosomes can effectively promote the proliferation and secretion capacity of dermal fibroblasts (Fang et al., 2019). Exosomes are also able to promote collagen formation in the extracellular matrix through its membrane surface LOXL2 protein (de Jong et al., 2016). Other identical studies revealed that exosomes could be isolated in fiber networks, demonstrating their involvement in the formation and functional regulation of extracellular matrices (Huleihel et al., 2016). In addition, exosomes can accelerate the repairing process and restrict scab size by interacting with the Annexin A1 and formyl peptide receptors to complete tissue remodeling (Nakamura et al., 2016b).

## Application Prospect and Open Question

### Exosomes and Scar Free Treatment

Skin wound healing is often accompanied by scab and scar formation, when the skin tissue is seriously damaged, exceeding its self-healing capacity, the wound area will be replaced by fibrous scars. The atrophy period of scar formation will bring physiological discomfort to the patient, and after the scar formation will certainly affect the local appearance and function. Our previous findings indicate that hucMSC-Ex retain YAP in the cytoplasm by transporting the active 14-3-3 molecule at the later stage of repairing, thus inhibiting the excessive proliferation of skin cells and the formation of fibrosis (Zhang et al., 2016). HucMSC-Ex also inhibit scar formation by inhibiting TGF- beta/SMAD2 pathway activation in fibroblasts by transshipping mir-23a, mir-125b and mir-145b, etc., thereby inhibiting myofibroblast activation (Chen J. et al., 2019). Meanwhile, AdMSC exosomes can reduce scar area and inhibit the differentiation of fibroblasts into myofibroblasts, mechanically related to the inhibition of TGF- beta 1 signaling and as well as ERK/MAPK signaling activation in fibroblasts (Wang et al., 2017). However, scars such as hypertrophic scars, resulting from alterations in the normal processes of cutaneous wound healing are finally featured by excessive deposition of collagen, persistent inflammation and fibrosis. New therapies including exosomes are designed to no more than minimizing scarring and accelerating wound healing immediately to avoid scar formation at an early stage. The hypertrophic scar treatments and even the keloid treatments still call for intensive studies.

### Exosomes as Bio-Markers for Cutaneous Diseases

Exosomes are carrying a variety of bioactive nucleic acids or proteins, which have been widely studied and reported as diagnostic markers of various diseases. Both the quantity and contents may alter when pathological conditions occur. In comparison with healthy controls, serum derived exosomes are more secretion in cutaneous diseases, especially tumors such as melanoma. Moreover, exosomes derived from melanoma patients are proved to convey an increased level of S100B (Alegre et al., 2016; Isola et al., 2016). Due to EV-related miRNAs are tissue-specific and exist in most biological fluids, they may serve as a tumor biomarker in early diagnosis, treatment response and prognosis of cancer (Mirzaei et al., 2016; Chen L. et al., 2019; Rahbarghazi et al., 2019). Exosomal miR-17, miR-19a, miR-21, miR-126, and miR-149 were increased in serum of patients diagnosed with melanoma (Chen L. et al., 2019). The expression level of exosomal miR-222 derived from melanoma samples was consistent with the metastatic capacity of melanoma cells (Felicetti et al., 2016). With the exception of skin tumors, the numbers of exosomes in the serum of patients with scleroderma is also remarkably higher than healthy group, which was of certain diagnostic value.

Recently, by using mass spectrometry and RNA sequencing, vast information has been acquired, which can be reference to the disease of interest. For example, profiles of neutrophils derived exosomes revealed 281 dysregulated lncRNAs and 22 dysregulated miRNAs as well as the predicted target genes and reactome pathways such as Wnt, IL-23, AMPK, and NOTCH signaling in systemic sclerosis, which could be high diagnostic value in the disease (Li L. et al., 2020). A mass spectrometry-based proteomic characterization of melanoma exosomes identified 1507 tumor-related proteins, most of which participated in cell proliferation, migration, EMT and neovascularization, which indicated a potential for further research identifying new biomarkers (Surman et al., 2020). Nevertheless, to be a competent diagnostic marker, more comprehensive case data and criteria are needed to promote its transformation from basic science to clinic. In order to realize the diagnostic and prognostic potential of exosomes containing distinct biological markers, it is essential to evolve the proper exosome isolation methods. Effective and clean without contamination separation of exosomes in circulation should be conducted to gain valid information for clinical application, the selected cargos should be specific and sensitive.

### Tailored Exosome as New Therapeutics for Cutaneous Disease

**FIGURE 5:**
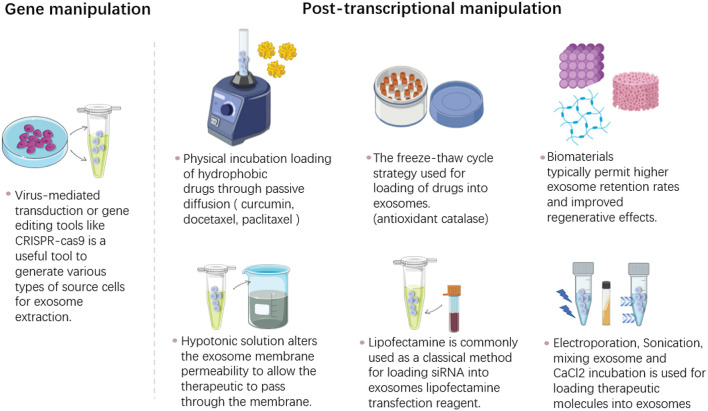
The bioengineering modification of exosomes. This is a simplified illustration of methods for modification of therapeutics into exosomes. The methods are basically divided into two parts, the gene manipulation modification and the post-translational modification. The first one is to use virus mediated system or gene editing techniques to modify the parental cells before exosome extraction. The other way is to directly modify exosomes after the isolation process by various method including incubation, electroporation, sonication, extrusion, hypotonic dialysis, freeze-thaw cycles, saponin, CaCl_2_ and lipofectamine reagent.

The positive role of exosomes in cutaneous wounds has been continuously recognized. At present, a craze of engineering modification of exosomes has been set off in the research field, which maximizes the advantages of exosome by gene manipulation of origin cells, drug or therapeutic molecular loading by physical incubation, the freeze-thaw cycling, electroporation, sonication and other techniques (Figure 5). The well-designed exosomes could erase the limitations of natural exosomes and increase the targeting capability, half-life in circulation as well as the cargo concentrations.

The endogenous way to get proteins encapsulated into exosomes is mainly by protein fusion which depends on the available cellular machinery for classifying therapeutic proteins into exosomes (Nasiri Kenari et al., 2020). Proteins such as lactadherin, lamp2b, which exist on the membrane of exosomes are used to deliver therapeutic siRNA, miRNA to the site of interest, tetraspanin family members CD9, CD63, CD81 are mostly used as a candidate for fusion of fluorescent proteins to trace exosomes *in vitro* and *in vivo*. In addition, tailored exosomes by transferring certain specific miRNA or siRNA has also been explored for disease treatment. By gene manipulation of synovial MSCs, exosomes express a high level of miR-126 to promote the angiogenesis in skin wound (Tao et al., 2017). Other pre-treatment of the origin cells like chemical agents, hypoxia is also effective for exosome modification. Melatonin-pretreated MSC-derived exosomes can enhance diabetic wound healing by regulating macrophage M1-M2 conversion (Liu et al., 2020). In our previous study, we found that small molecule drugs 3,3′-diindolymethane (DIM), as a natural small-molecule drug extracted from cruciferous plants, can activate hucMSC-Ex and enhance the dry and paracrine ability of MSC by carrying active Wnt11 molecules, so as to enhancing the repairing effect of deep second-degree scald wounds (Shi H. et al., 2017).

Exosomes also can be modified exogenously after their isolation process. Approaches including electroporation, incubation, sonication, extrusion, hypotonic dialysis, freeze-thaw cycles, saponin, CaCl_2_ and lipofectamine reagent are used for exosomes loading. Sources such as platelet derived exosomes loaded with curcumin on polysaccharide, can more effectively promote diabetic foot ulcer wound repair (Brennan et al., 2020). MSC derived exosomes assembled in a chitosan/silk hydro gel (Shiekh et al., 2020) and in a self-healing, anti-bacterial polypeptide hydro gel (Wang et al., 2019) promoted diabetic wound healing. It can be predicted that exosome modification will make it play its maximum advantages in cutaneous wound healing. Meanwhile, it remains to be determined the specific mechanism of cargo loading, release, uptake, and destiny of vesicle in recipient tissue.

### Future Application Prospects and Problems of Exosomes

Exosomes can significantly repair cutaneous damage, provide new therapeutic strategies for the treatment of various diseases, and lay an important foundation for biopharmaceuticals based on exosome modification. However, there still exists many unanswered questions for exosome utilization from basic science to clinic: (1) The scalability of exosomes is a major challenge currently. The future clinical use for exosomes depends on a mass-produced and GMP-compliant production process. New approaches like tangential flow filtration can concentrate large quantities of cell-conditioned media for exosome enrichment (Heinemann et al., 2014). (2) There is an urgent need for precise and accurate characterization of exosomes to better evaluate their heterogeneity, their cargo, and functions. A new criterial for exosome identification is put forward by MISEV (Théry et al., 2018). (3) The use of supraphysiological numbers of exosomes still needs to be re-considered, for example, the dose, time duration as well as the route of administration. (4) It is still unclear that how the multiple information conveyed by exosomes can be fully used to develop more precise or early-phase biomarker to help clinical decision making. (5) The application of exosomes as therapeutics relies on producing homogeneous exosomes on a large scale by finding optimal producer cells, culture conditions as well as stimulation conditions, subsequently, getting the approval by the FDA for clinical applications. A large amount of studies has demonstrated the role of exosomes, especially MSC-exosomes in tissue regeneration, immunomodulation and tumor. However, exosomes under different conditions may have opposite effect, for example, exosomes from aged MSC shows no effect on skin regeneration. Further investigation is still required to uncover the multifaceted function of exosomes.

Ultimately, although we have already acquired certain stock of knowledge on exosomes and their functions on skin biology, there still much room for exploration on the potential of exosomes in dermatologic disease is diagnose and treatment.

## Author Contributions

HS did the conceptualization. HS and YS wrote. MW and DY collected the data. HQ and WX reviewed and edited. All authors contributed to the article and approved the submitted version.

## Conflict of Interest

The authors declare that the research was conducted in the absence of any commercial or financial relationships that could be construed as a potential conflict of interest.

## Publisher’s Note

All claims expressed in this article are solely those of the authors and do not necessarily represent those of their affiliated organizations, or those of the publisher, the editors and the reviewers. Any product that may be evaluated in this article, or claim that may be made by its manufacturer, is not guaranteed or endorsed by the publisher.

## Abbreviations

MVB: multivesicular body
MSC: mesenchymal stem cells
ILV: intraluminal vesicles
EV: extracellular vesicle
ESCRT: endosomal sorting complex required for transport
TSG101: tumor susceptibility gene 101 protein
ALIX: ALG-2 interacting protein X
VPS: vacuolar protein sorting-associated protein
SNARE: soluble NSF Attachment Protein Receptor
DPC-Ex: dermal papilla cells derived exosomes
MAPK: mitogen-activated protein kinase
EMT: epithelial-to-mesenchymal transition
MSC: mesenchymal stem cell
hucMSC: human umbilical cord derived MSC
BM-MSC: bone marrow derived MSC
hiPSCs: human induced pluripotent stem cells
MMPs: matrix metalloproteinases
3D: three-dimensional
TIMP-1: tissue inhibitor of metalloproteinases-1
SHSs: sponge sp. spicules
DFU: diabetic foot ulcer
GLUT: glucose transporter
MDSC: myeloid-derived suppressor cell
SCC: squamous cell carcinoma
TF: tissue factor
IGF-1: insulin-like growth factor-1
IL-6: interleukin-6
VEGF: vascular endothelial growth factor
HSP90α: heat shock protein 90α
AdMSC: adipose derived MSC
DIM: 3,3 ′-diindolymethane
MISEV: minimal information for studies of extracellular vesicles
FDA: Food and Drug Administration.
